# Quantification of Food Intake in *Drosophila*

**DOI:** 10.1371/journal.pone.0006063

**Published:** 2009-06-26

**Authors:** Richard Wong, Matthew D. W. Piper, Bregje Wertheim, Linda Partridge

**Affiliations:** 1 Institute of Healthy Ageing, and GEE, University College London, London, United Kingdom; 2 Evolutionary Genetics, University of Groningen, Groningen, Netherlands; Queen Mary College, University of London, United Kingdom

## Abstract

Measurement of food intake in the fruit fly *Drosophila melanogaster* is often necessary for studies of behaviour, nutrition and drug administration. There is no reliable and agreed method for measuring food intake of flies in undisturbed, steady state, and normal culture conditions. We report such a method, based on measurement of feeding frequency by proboscis-extension, validated by short-term measurements of food dye intake. We used the method to demonstrate that (a) female flies feed more frequently than males, (b) flies feed more often when housed in larger groups and (c) fly feeding varies at different times of the day. We also show that alterations in food intake are not induced by dietary restriction or by a null mutation of the fly insulin receptor substrate *chico*. In contrast, mutation of *takeout* increases food intake by increasing feeding frequency while mutation of *ovo^D^* increases food intake by increasing the volume of food consumed per proboscis-extension. This approach provides a practical and reliable method for quantification of food intake in *Drosophila* under normal, undisturbed culture conditions.

## Introduction

The fruit fly *Drosophila melanogaster* is a key model organism for discovery of evolutionarily conserved biological mechanisms, which include the control of nutrient sensing [1], [2], feeding [3]–[5] and ageing [6], [7]. Reliable methods for measuring food intake of *Drosophila* are therefore often needed. However, quantification of food consumption in the fly poses challenges. In mammals, food ingestion can be directly quantified by weighing the food before and after feeding has taken place. However, flies consume volumes of food that are too low to weigh accurately, and feed by extension of their proboscis into the food medium, prohibiting direct observation of the volume of food ingested. One method has overcome this problem by measuring the food consumed in liquid form in a capillary feeder (CAFE) [8]. However, despite being effective for quantifying intake, CAFE feeding substantially reduces both the egg-laying and lifespan compared to those seen in flies provided with food in the usual agar-gelled medium [7], [9]. This may be because in nature *Drosophila* feed on microorganisms, particularly yeast, on the surface of fruit [10], [11], and thus feeding on a liquid diet from a capillary may not reflect their natural feeding environment.

To overcome the problems of measuring food intake when flies feed on gelled media, several studies have made indirect measures of food uptake after marking the food, either with a visible dye [12]–[14] or with radioactively-labelled nutrients [15]–[17]. However, such ‘tracer’ methods have limitations and can even give misleading results. Transferring flies to labelled food creates a disturbance that could change the volume of food ingested per proboscis-extension (ingestion ratio) and/or the frequency of proboscis-extension (feeding frequency), and therefore measurements immediately after transfer may not be an accurate reflection of food consumed during undisturbed conditions. Furthermore, because tracer methods rely on measuring only the volume of label present in the fly, the results can be influenced by factors other than feeding, and substantial differences in either the ingestion ratio or feeding frequency can be over-looked [18]. For instance, if the internal capacity of the flies for the label is increased by the experimental treatment, with no alteration in feeding, then with increasing times of exposure to the labelled food, the group with the higher internal capacity will give the spurious appearance of having a higher food intake. This problem can occur with dietary restriction in *Drosophila*, which increases the capacity of the crop [18]. In addition, if flies differ in food intake but not in internal capacity for the food tracer, then once steady state is reached with rate of egestion of the label equalling the rate of intake, the amount of label present in the two groups of flies will be the same, despite their difference in food intake [18]. For the amount of label in the fly to reflect feeding, measurements must therefore be confined to the time period before label egestion commences, about 40 minutes in *Drosophila*, a time period during which disturbance of the flies affects their feeding behaviour. There is thus a requirement for a method of measuring feeding in undisturbed conditions.

Previously, we have reported that direct observations of fly proboscis-extension onto the food surface [19] can indicate food intake. This assay offers three advantages over the methods mentioned above: 1) repeated assays can be performed with the same flies through time because no flies are sacrificed for measurements, particularly valuable in the context of work on ageing; 2) the observations can be made on flies housed on standard laboratory food, and could be extended to other culture conditions; 3) food intake can be measured during undisturbed conditions once the proboscis-extension observations are calibrated by measures of short-term dye-accumulation, to determine the volume of food ingested per proboscis-extension.

In this study we tested the accuracy of the method, by measuring the volume of food ingested using a dye food label in parallel with observing the number of proboscis-extensions. We compared flies that had either known or suspected differences in food intake, such as males versus females, flies subjected to dietary restriction [20] and *chico* ^1^ [21], *takeout* ^1^ [22] and *ovo* ^D1^ [23] mutant flies relative to their controls. Additionally, we also checked that the ingestion ratio did not alter with age, by performing the combined assay on flies over various days of a lifespan.

Dietary restriction (DR) in *Drosophila* is often achieved by dilution of the food medium, and complete records of food intake are needed to determine if flies compensate for the reduced nutritional content of food by increasing the total amount of food they consume. Measurement of food intake is also needed to determine if other interventions, such as sensory perception of food [24], or reduced insulin/insulin-like growth factor signalling (IIS) [6], [25] extend lifespan by reducing nutritional intake and hence act by inducing a state of DR. We tested this possibility by measuring the food intake of flies carrying a mutant for the IIS gene *chico*, which extends the lifespan of *Drosophila* [21].

## Results

### Establishing a relationship between proboscis-extension and total volume of food eaten

In nature and in the laboratory, fruit flies feed on the food surface, by extending their proboscis into contact with the food and drawing it in. The amount of proboscis-extension onto the food surface was measured by making periodic observations of groups of flies. The number of observations of proboscis-extension was then expressed as a proportion of the total number of observations [19]. Short-term food consumption was quantified by transferring flies onto food labelled with a non-toxic, non-absorbed blue dye. The amount of blue food present in the fly was quantified using spectrophotometry [12]. The assay period was confined to the 30 minutes after transfer, because the dye is egested shortly after this length of time [18]. Thus a 30-minute exposure period to blue dyed food ensured that all dyed food eaten during the assay is retained in the fly gut and none was lost by egestion.

**Figure 1:**
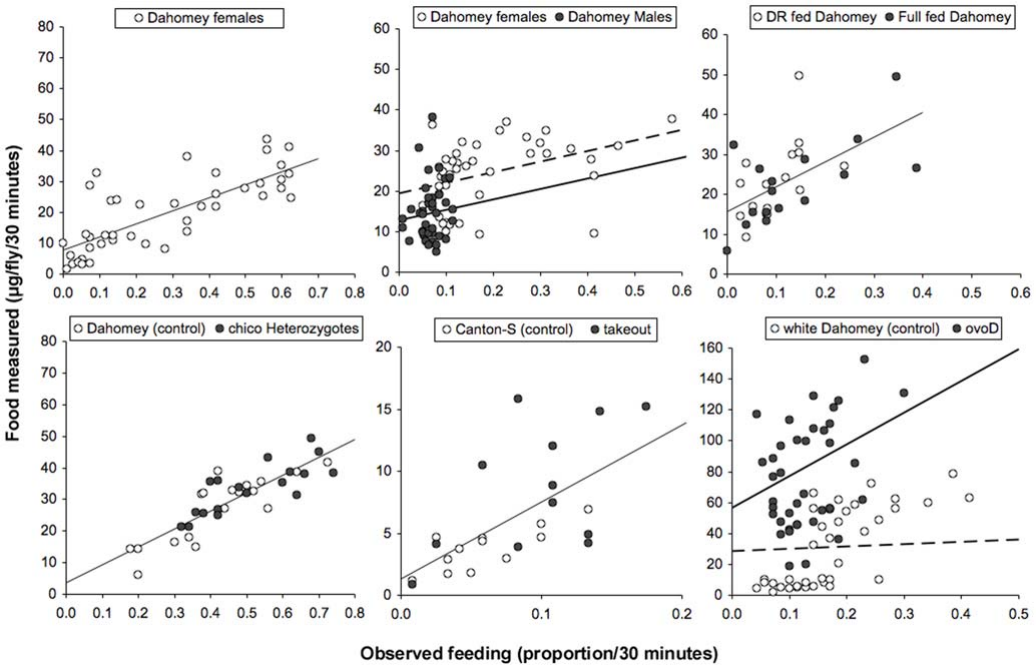
Measurements of blue label uptake after 30 minutes of feeding and the proportion of feeding events observed during this period, where one circle represents one vial containing 5 flies. Trend lines represent the relationship between the volume of food ingested and the observed proportion of flies feeding (V/O) described in Table 1. Dashed lines represent open circles. All flies were female unless stated, were 7 days old and were allowed to mate for 48 hours after eclosion (NF = the number of flies per condition, NV = the number of vials per condition). (a) A linear (V/O) relationship existed in mated Dahomey females (NF = 210, NV = 42). (b) The V/O relationships of mated Dahomey males and females did not differ significantly, although females were found to have fed at a greater frequency than males during the 30 minutes (NF = 200, NV = 40). The gradient for males did not differ significantly from that for females but had a lower intercept. (c) DR fed and full fed Dahomey females shared the same V/O relationship and no difference in feeding between dietary conditions was found with the combined assay (NF = 75, NV = 15). (d) The V/O relationship was the same in *chico* ^1^ heterozygotes and in the Dahomey control. No difference in feeding between genotypes was found with the combined assay (NF = 90, NV = 18). (e) The V/O relationship was the same in *takeout* ^1^ and in Canton-S females, even though *takeout* ^1^ flies were found to feed at a higher frequency than Canton-S controls (NF = 60, NV = 12). (f) Both *ovo* ^D1^ and *white* ^Dahomey^ females had a positive V/O relationship, but *ovo* ^D1^ flies had a significantly greater gradient and intercept, and therefore increased the volume of food ingested per proboscis-extension more quickly than *white* ^Dahomey^ females (NF = 200, NV = 40).

To compare proboscis-extension measurement against dye ingested, we performed the two assays described above on the same cohort of flies. Initially, groups of 5, 7-day-old mated female flies were allowed to feed for 30 minutes on food labelled with blue dye [12] while we simultaneously observed the proportion of time they spent with the proboscis extended [19]. Flies were then sampled, and the amount of blue food they ingested quantified. We then plotted the level of blue food measured in the group against the proportion of proboscis-extensions observed in that group (**Figure 1a**). We found a strong positive linear relationship between the Volume of blue food found in the fly and the proportion of feeding events Observed (V/O) (*P*<0.0001, linear mixed effect model, LMEM). The gradient of this relationship represents the ingestion ratio of the flies, as it describes the volume of blue accumulated per proboscis-extension. To test for non-linearity (for example, saturation or acceleration in the V/O relationship), we added a quadratic term to the statistical model. The quadratic term was not significant (*P* = 0.62), indicating the V/O relationship is indeed linear over the timespan we measured (**Table 1**). The linear relationship demonstrated that the proboscis-extension method is an accurate indicator of food intake in female *Drosophila* under these conditions.

**Table 1:** A linear relationship was tested between blue dye accumulations and feeding frequency using ANOVA in linear mixed effects model.

Assay	Fixed effects	P-value	V/O relationship		
			Coefficient	Estimate	Standard error
Dahomey females (NF = 210, NV = 42, 5 trials)	Observation	<0.0001	Intercept	7.91	2.50
			Gradient	42.10	7.99
Dahomey males vs Dahomey females (NF = 200, NV = 40, 4 trials)	Observation	<0.0001	Intercept F	19.53	2.82
	Sex	<0.001	Intercept M	12.83	1.80^b^
	Observation∶Sex	not sig.	Gradient	25.81	8.58
Fully fed vs Dietary restriction (NF = 75, NV = 15, 1 trial)^a^	Observation	<0.001	Intercept	15.68	2.40
	Diet	not sig.	Gradient	62.25	16.11
	Observation∶Diet	not sig.			
*chico* heterozygous vs Dahomey control (NF = 90, NV = 18, 3 trials)	Observation	<0.0001	Intercept	4.50	2.96
	Genotype	not sig.	Gradient	55.04	6.02
	Observation∶Genotype	not sig.			
*takeout^1^* vs Canton-S (NF = 60, NV = 12, 1 trial)^a^	Observation	<0.001	Intercept	1.29	1.25
	Genotype	not sig.	Gradient	62.01	15.78
	Observation∶Genotype	not sig.			
*ovo^D^* vs *white^Dahomey^* (NF = 200, NV = 40, 4 trials)	Observation	<0.0001	Intercept *ovo^D^*	56.40	16.48
	Observation∶Genotype	<0.001	Intercept *w^Dah^*	28.65	8.60^b^
	Genotype	<0.0001	Gradient *ovo^D^*	205.52	37.22
			Gradient *w^Dah^*	14.46	8.94

The *P* value of the interaction terms is also displayed, which indicated whether the regression coefficients differ between comparative conditions (NF = no. of flies per condition and NV = no. of vials per condition).

a These assays were not repeated on different trial dates. The statistical analysis was therefore only on fixed effects, i.e., a regression analysis.

b These standard errors are for the differences in the intercepts.

Next, we tested whether the sexes differed in ingestion ratio (gradient of the V/O relationship) by repeating the combined assay with 7-day-old mated males and females (**Figure 1b**). The ingestion ratio was constant in males and in females, as both were found to have a significant V/O relationship (*P*<0.0001, LMEM). The gradients of these relationships were not found to be significantly different (*P* = 0.9871), indicating that the ingestion ratio did not differ between the sexes. However, the intercept of the male relationship was significantly lower than that for females (*P*<0.001) and suggested males across all observations contained a lowered basal level of blue dye content than in females (**Table 1**). This could be due to differences in body size and/or body composition (e.g., proportions fat, muscles and reproductive tissues). As in the previous analysis, the quadratic term was not significant (*P* = 0.54), indicating that a linear V/O relationship exists. In spite of the sexes sharing the same ingestion ratio, females were found to have fed more than males over the 30-minute period because they spent a greater proportion of time with the proboscis extended (2.8-fold more on average) than males (*P*<0.0001, generalised linear model, GLM). This suggested it is possible for flies to increase their food intake by feeding at a greater frequency rather than by consuming in greater volume, and it is possible to detect such differences in food intake.

We then extended the use of this method to examine the effect of other factors that could determine the physiology and behaviour of feeding flies. The nutritional environment may be such a factor, and is particularly important in the context of DR experiments where dietary dilution is employed to restrict access to nutrients. We therefore performed the combined assay with 7-day-old mated females that were fed either DR or full fed control diet [7] (**Figure 1c**). Flies on differing yeast concentrations did not alter the ingestion ratio, because no significant difference in V/O relationship was found (*P*<0.0001, linear regression model), with no significant differences in the gradient and intercept of this relationship between the two different diet regimes (*P* = 0.447, respectively, *P* = 0.304: **Table 1**). Flies on the DR diet were also found not to compensate for the reduced nutrient availability by feeding more often, because the proportion of proboscis-extensions between DR fed and full fed flies during the 30-minute period of the combined assay were not different either (*P* = 0.3693, GLM).

Finally, we also tested whether the ingestion ratio or feeding frequency were altered by genetic mutations known or suspected to affect feeding. The first mutation, *chico^1^*, is a null mutation in the single fly insulin receptor substrate in the insulin/insulin-like growth factor-1 signalling (IIS) pathway, a pathway suggested to affect foraging and feeding in larvae [26]. We performed the combined feeding assay on 7-day-old mated female heterozygotes of *chico^1^* and their genetic control (Dahomey) (**Figure 1d**). The ingestion ratio did not differ between *chico^1^* heterozygotes and their controls, because a significant V/O relationship exists (*P*<0.0001, LMEM), with no significant differences in the gradient or intercept between *chico^1^* heterozygotes and control flies (*P* = 0.3177, respectively, *P* = 0.3947, **Table 1**). *chico^1^* heterozygous flies and their controls had the same food intake, because the proportion of proboscis-extensions between the cohorts during the 30-minute period of the combined assay were also not significantly different (*P* = 0.0831, GLM).

The second mutation, *takeout^1^*, is in a gene reported to regulate the circadian rhythm and to increase food intake prior to starvation in *Drosophila* [22]. We performed the combined feeding assay on 7-day-old mated *takeout^1^* flies and their genetic control (Canton-S) (**Figure 1e**). The ingestion ratio did not differ between *takeout^1^* flies and controls, because a significant V/O relationship existed (*P*<0.0001, linear regression model) with gradient and intercept not significantly different between the two genotypes (*P* = 0.5931, respectively *P* = 0.0549: **Table 1**). This suggested that the ingestion ratios in both *takeout^1^* flies and controls were similar. However, *takeout^1^* flies fed more than controls, because they spent 1.6-fold more time with their proboscis extended on the food than did Canton-S flies (*P*<0.05, GLM). The flies thus elevated their nutrient-intake by feeding at a greater frequency, rather than by increasing the volume of intake per proboscis-extension.

The final mutant studied, *ovo* ^D1^, causes female sterility and has been reported to induce a reduced feeding frequency [23]. We performed the combined assay with 7-day-old, mated, mutant females and their genetic control (*white* ^Dahomey^) (**Figure 1f**). A significant V/O relationship was found for both cohorts (*P*<0.0001, LMEM); however, the gradient and the intercept for the relationship differed between the two genotypes (*P*<0.0001 and *P*<0.001, respectively). The V/O gradient for *ovo* ^D1^ was steeper (205.52 versus 14.46 in *white* ^Dahomey^) and the intercept greater (56.40 versus 28.65 in *white* ^Dahomey^) than for *white* ^Dahomey^ controls (**Table 1**). *ovo* ^D1^ females thus had ingested a greater volume of food per proboscis-extension compared to *white* ^Dahomey^ controls (accumulated blue dye faster with each proboscis-extension), as well as a greater basal level of blue dye. However, no significant difference in the proportion of time spent feeding between *ovo* ^D1^ females and *white* ^Dahomey^ controls was recorded (*P* = 0.6289, GLM). This indicated that *ovo^D1^* flies elevated their received nutrition by increasing the volume of intake per proboscis-extension rather than by feeding at a greater frequency.

**Figure 2:**
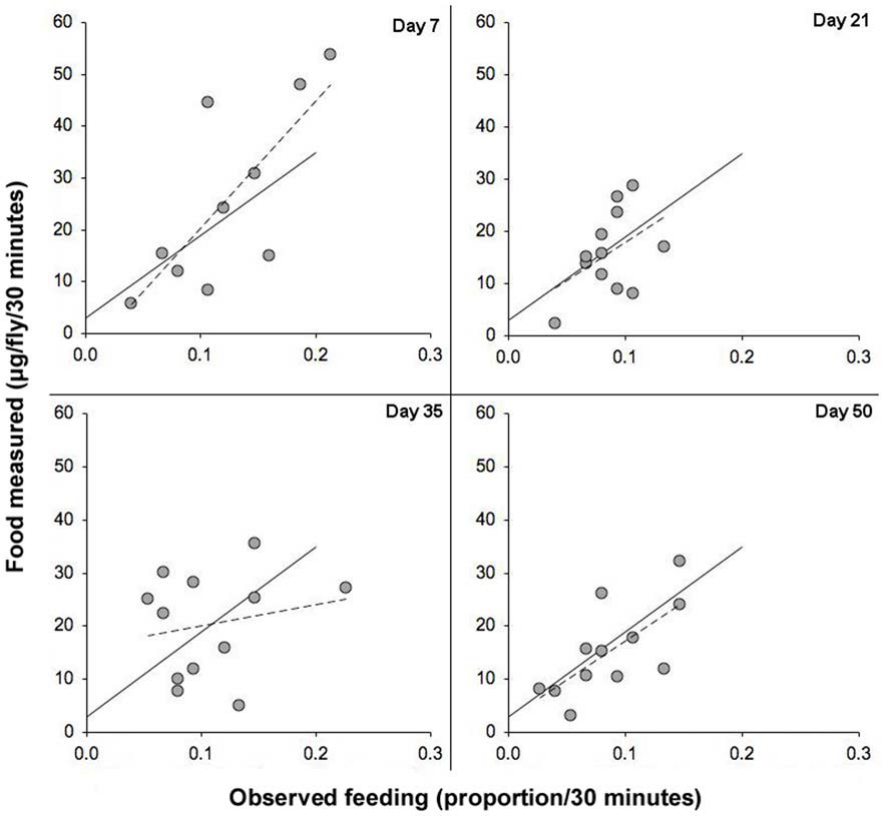
The relationship between blue label uptake and observed feeding events did not change for flies of advancing age. Circles represent measurements of blue label uptake after 30 minutes of feeding and the proportion of feeding events observed during this period. One circle represents one vial containing 5 flies. Experiments were conducted with mated Dahomey females. Assays occurred at 4 different ages: on days 7, 21, 35 and 50 after eclosion. Each assay used 60 flies (12 vials) that were taken from a population that began with 500 individuals. Solid lines represent the significant (*P*<0.0001) V/O relationship with a gradient coefficient of 160.36 (S.E. = 31.39) and intercept of 2.89 (S.E. = 3.45), dashed lines represent the line of best fit for each age class.

We also analysed the effect of age upon the ingestion ratio. Dahomey females were subjected to the combined blue dye and proboscis-extension assay at 4 different ages (day 7, 21, 35 and 50: **Figure 2**). The V/O relationship was highly significant at all ages (*P*<0.0001, linear regression model), but neither the gradient (*P* = 0.0961) nor the intercept (*P* = 0.649) changed with age. The volume of intake per proboscis-extension was thus unaffected by the age of the flies.

During the 30 minutes of the combined assay, the flies consumed amounts of blue label that spanned a 30-fold range (equivalent to that found in 5 µg–150 µg food). The food intake of the flies thus varied widely. Despite the variation in the overall amount of feeding, there was no significant variation in the ingestion ratios except in one genotype, *ovo* ^D1^. The variation in observed food intake is a possible indicator that transferring flies to labelled food may temporarily disturb their feeding behaviour and highlights the importance of measuring feeding during undisturbed conditions if a quantitative measure of normal intake is required. In addition, control-feeding frequency must be measured at the same time as that in the experimental treatments.

### Factors that influence feeding during undisturbed conditions

We investigated other variables that could affect food intake during undisturbed conditions. The circadian rhythm is reported to alter feeding in *Drosophila* [27], there may also be an effect from differences in group size, either in a positive (e.g. aggregation behaviour [28]) or negative (e.g. aggressive competition [29]) direction, and finally, dietary composition may also affect feeding.

**Figure 3:**
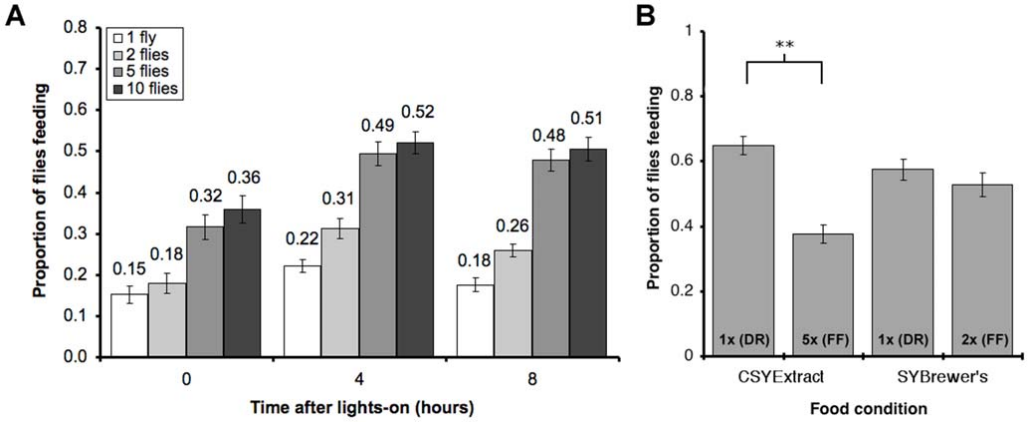
Possible factors that influence feeding frequency. (a) The proportion of time spent feeding of 7-day old mated females over a 2-hour period at varying times after lights-on. Females were housed alone, or in groups of 2, 5 or 10 (the number of flies for each condition = 30, with 30 vials for single flies, 15 vials for groups of 2, 6 vials for groups of 5 and 3 vials for groups of 10). We found that increasing the number of flies per vial increased the feeding frequency of each fly, and overall, flies fed more frequently in the afternoon and evening. We calculated the proportion of time spent feeding by summing the scored feeding events divided by the total number of feeding opportunities, which is unaffected by the difference in the number of vials per condition (b) The proportion of time spent feeding for flies fed different yeast-based diets. Flies were fed two similar diets containing either a water-soluble yeast extract (CSYExtract) or lyophilised yeast (SYBrewer's) at two different concentrations (DR = Dietary Restriction, FF = Full Fed). While feeding frequency was sensitive to the concentration of yeast extract in the diet, it was unchanged by the concentration of lyophilised yeast (NF = 60 and NV = 12 per condition: ** = *P*<0.005, and error bars = S.E.).

To test these factors, we performed the undisturbed proboscis-extension assay at 3 different times in the day. Flies are maintained in a 12h∶ 12h light∶ dark cycle, and lights-on occurs at 10am and lights-off occurs at 10pm. We performed the proboscis-extension assay in the morning (at lights-on), in the afternoon (4 hours after lights-on), and in the evening (8 hours after lights-on) using 4 different group sizes (1, 2, 5 or 10 flies: **Figure 3a**). Both the time of day and the group size had highly significant effects on the proportion of time spent feeding (*P*<0.001 for both group size and time of day, GLM), while the interaction between these two was not significant (*P* = 0.88). The lowest feeding proportion was observed in the morning for flies housed singly (0.15 of the time spent feeding), and this increased to approximately 0.50 in the afternoon and evening for flies feeding in groups of 5 or more. Both the afternoon and evening feeding proportions were significantly higher than those in the morning (*P*<0.0001 in both cases, GLM). There was no significant difference in feeding proportions between flies during the afternoon and evening (*P* = 0.182, by model simplification). The lowest proportion of feeding was observed for flies housed singly 0.15–0.22 (depending on time of day), and this significantly increased to 0.18–0.31 (depending on time of day) when flies were housed in pairs (*P* = 0.009, GLM). The proportion of flies feeding was found to nearly double when the number of flies was increased to 5 per vial (0.32–0.49, depending on time of day; 2 flies per vial against 5 flies per vial, *P*<0.0001, GLM), and did not increase further when flies were housed at 10 per vial (0.36–0.52, depending on time of day; 5 flies per vial against 10 flies per vial, *P* = 0.287, by model simplification: **Figure 3a**).

Finally, we tested the response of 7-day old female flies to two different yeast-based diets, one made with water-soluble yeast extract (CSYExtract) [15] and the other with lyophilised yeast (SYBrewer's) [7]. The principle difference between these diets is that yeast extract contains only the water-soluble portion of an autolysed yeast culture, whereas the Brewer's yeast product is made of all cell contents and debris after autolysis and pasteurisation. Both of these have previously been used to study the effects of DR [7], [15] (**Figure 3b**). The foods 5× CSYExtract and 2× SYBrewer's represent full-fed (FF) conditions, while 1× CSYExtract and 1× SYBrewer's represent DR conditions. The food composition had a significant effect on feeding frequency (*P* = 0.0126, GLM). As previously reported, flies exhibited significantly lower feeding frequency when the concentration of yeast extract was increased in the CSYExtract diet (1× CSYExtract against 5× CSYExtract, *P* = 0.0019, GLM). In contrast, the feeding frequency of flies was unaffected when altering the yeast concentration of the SYBrewer's diet (1× SYBrewer's against 2× SYBrewer's, *P* = 0.562, GLM).

### Measuring food intake in lifespan studies

The proboscis-extension method allows repeated feeding assays to be performed with the same cohort of flies, an advantage over methods that sacrifice flies during measurements. As far as we are aware, no publication to date has studied either the feeding frequency of a cohort of flies throughout their lifespan or measured how much food flies consume throughout their lives. This is especially important when monitoring the effects of dietary restriction on lifespan, as the short-term probability of death as revealed by mortality analysis is rapidly affected by changes in nutritional conditions [30]. Thus feeding data from a single time point early in life may not be informative about DR because they do not reflect nutrient intake changes that could occur close to the time of death.

**Figure 4:**
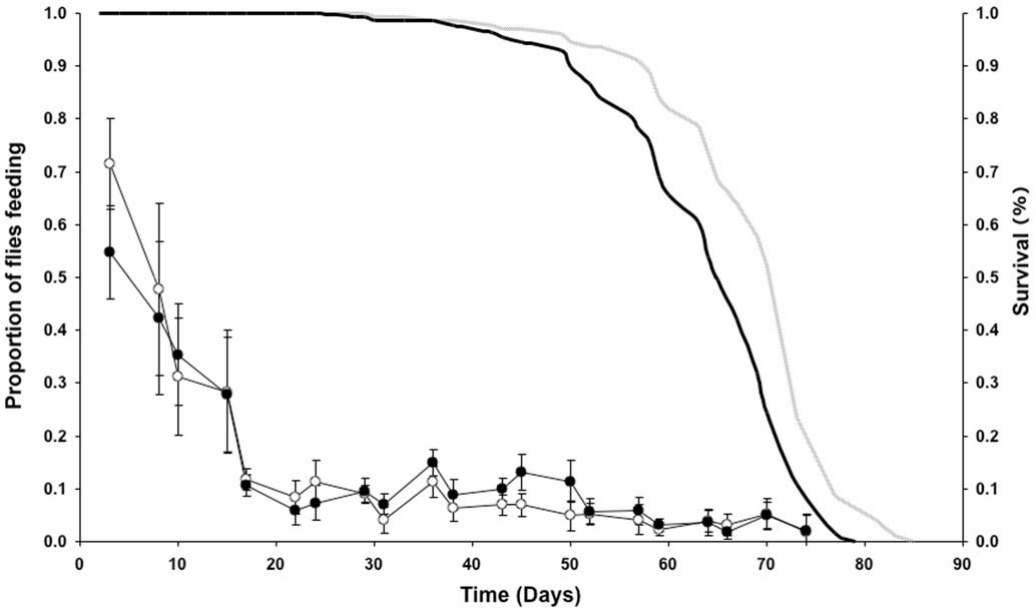
The proportion of time spent feeding for DR (open circles) and full fed (FF) flies (closed circles) on different days of their lifespan. Survivorship curves are indicated with a solid grey line (DR) and a solid black line (FF) flies. Median lifespan: DR = 70 days, FF = 65 days. Proboscis-extension assays used 150 flies (30 vials) per condition. Flies were maintained in populations that began with 1500 individuals per condition (error bars = S.D.).

**Figure 5:**
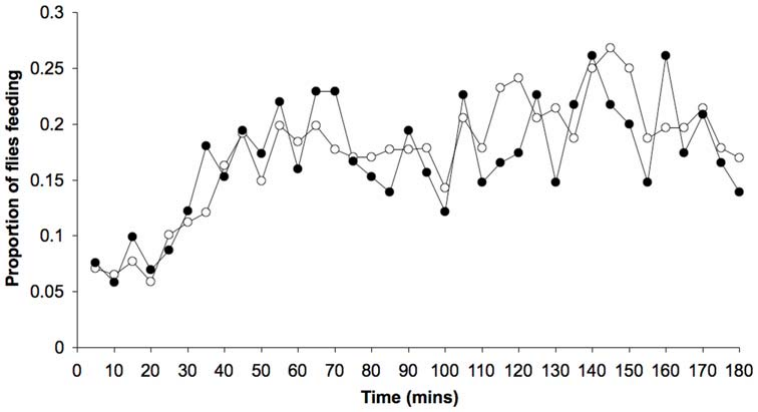
The proportion of time spent feeding during a proboscis-extension assay for DR (open circle) and fully fed (closed circle) once-mated 14-day old females. Flies were maintained on different diets throughout their lifespan. DR females did not differ from fully fed females in feeding frequency. The assay began immediately when the observer arrived. Note the lower proportion of flies feeding during the first 30 minutes of the assay, which may relate to the appearance of the observer in the room (NF = 100; NV = 20).

We therefore compared the feeding frequency of once-mated females subjected to DR or control feeding over the course of their lifespan (**Figure 4**). We performed the proboscis-extension assay on cohorts of flies that were kept in a pooled population and assays were performed independently over their lifespan. Feeding declined markedly with the age of the flies, especially during the first 3 weeks of life. The changes in feeding frequency across the lifetime of the flies were significantly different on the two diets (significant interaction between Age and Diet, *P*<0.001, GLM). No overall difference was found in average feeding frequency (0.17 in both cohorts) for the course of the lifespan. However, flies on a DR diet fed in a greater proportion of observations than full fed flies early in life, while this reversed later in life when full fed flies fed more than DR flies (between day 31 and day 50), after which the feeding became similar on the two diets. Preliminary studies showed that the feeding frequency of flies on both diets were low at the beginning of the proboscis-extension assay but gradually increased to a steady state over 30 minutes (**Figure 5**).

**Figure 6:**
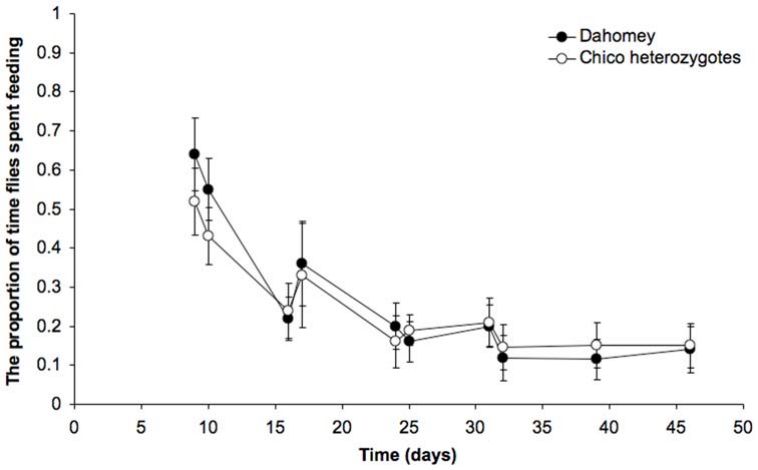
The observed proportion of time spent feeding for Dahomey (control) flies (closed circles) and *chico* ^1^ heterozygotes (open circles) on different days of their lifespan, obtained by dividing the number of flies observed feeding by the total number of flies present. Two observers alternately performed assays on the same population of flies. No significant interaction (*P* = 0.151) between the observers' data was found. Assays used 50 flies (10 vials) per condition, flies were maintained in populations that began with 500 individuals per condition; error bars = S.D.

We also compared the feeding frequency of wild type and long-lived *chico^1^* heterozygote flies over their lifespans [21]. Reduced *chico^1^* signalling could lead to a reduction in food intake at some period of life, and therefore increased lifespan through self-imposed DR. Analysis of proboscis-extension over lifetime found that *chico* ^1^ heterozygotes fed no more or less than Dahomey at any stage of their lifespan (*P* = 0.1639, GLM). Overall observed feeding proportions also did not differ significantly from wild type controls (*chico* ^1^ heterozygotes = 0.259 and Dahomey = 0.283, *P* = 0.3193, GLM: **Figure 6**). As observed before, feeding frequency declined markedly with the age of the flies for both genotypes, and this proved to be significant (*P*<0.001, GLM).

## Discussion

In this study, we validated an indirect method of measuring food intake in *Drosophila* (measuring proboscis-extensions) by combining it with a direct method (measuring food intake with a food dye). Despite considerable variation in feeding between replicate groups of flies and between experiments performed on different days, the volume of food ingested per proboscis-extension (ingestion ratio) did not significantly differ between females and males, flies of different ages, flies subjected to DR and flies with mutations in *chico* or *takeout*. Only *ovo* ^D1^ females ingested more dye per proboscis-extension.

The observation data revealed that males feed less than females. The higher food intake of female flies is presumably related to their high nutrient-usage in egg-production [31]. The difference in intercept between the two sexes in the combined measurement indicates that amounts of blue dye are always lower in males, although the increase in blue food per proboscis-extension is the same. These lower basal levels of dye may be due to the differences in size (the total volume of the crop and gut), or because the differences in body composition (e.g. fat tissue, vitellogenic material or muscles) may affect the spectrometer reading.

Sterile *ovo* ^D1^ females exhibited a greater ingestion ratio than any of the other genotypes tested. This finding is surprising, because egg development is arrested in *ovo^D1^* flies before the major nutrient investment occurs [32]. If the larger volume of food ingested reflects greater nutrient absorption and utilization in *ovo* ^D1^ flies, it could be that they expend more energy through a higher level of activity than fertile flies. This could partially explain why we found a higher ingestion ratio in these mutant flies. *ovo* ^D1^ has been reported as feeding less frequently during long-term undisturbed conditions [23], however, our results may not contradict those of Barnes *et al.* (2008), because our data were obtained from the first 30 minutes after transferring to blue-labelled food, and we found no differences with feeding frequency, only with ingestion ratio.

The combined assay is not suitable for long-term, undisturbed feeding experiments because the assay requires that flies are transferred to dyed food, which disturbs fly feeding behaviour. Frequencies of proboscis-extension were observed to be lower than in steady-state conditions, and only reached a constant level by the end of the 30-minute observation period. However, the indirect method alone is accurate for measuring fly feeding during long-term, undisturbed, experimental conditions once assessed by the combined assay for any differences in the ingestion ratio.

Data from the undisturbed steady-state studies suggested that flies exhibit marked diurnal differences in feeding behavior when feeding in groups and earlier in life. The effect of fly group size may reflect the role of aggregation pheromones, which act as communication signals between flies on breeding substrates, with feeding and oviposition rates increasing with the level of aggregation pheromone [28].

Mutations in the IIS pathway have been shown to extend the healthy lifespan of the nematode worm *Caenorhabditis elegans*, as well as *Drosophila* and the mouse [6], [33]–[35]. Hence, there is intense interest in understanding how the effects of this pathway on healthy lifespan are mediated. A possible cause for the lifespan-extension effect in flies is that they reduce their food intake, resulting in self-imposed DR. If true, this could also account for the observed overlap between the effects of altered IIS and DR in *Drosophila* [36]. Null mutation of the gene encoding the insulin receptor substrate *chico* in *Drosophila* both extends lifespan [21] and alters the response to DR [37]. We assessed the ingestion ratio and the undisturbed, long term feeding frequency of long-lived c*hico*-heterozygotes using the proboscis-extension assay and found total food intake was not reduced in the mutants. The increased survival of *chico^1^* mutant flies compared to controls can therefore not be explained by a reduction in food intake [21]. Thus the observed extension in lifespan in *chico^1^* mutants [21] is not simply due to self-imposed DR [30].

DR in flies can be imposed by dilution of their food source, which is available in excess. Flies could therefore adjust their feeding frequency to compensate for the reduction in nutritional value, thus reducing or eliminating the effect of food dilution on nutrient-intake. The literature on this topic is conflicting, with some reports that flies can partially compensate for the food dilution [15], others that they do not [18], [19] and others that even report increased food intake with increased nutrition [14]. Although each of these studies examined the effects of DR, none of them employ the same dietary conditions as each other. We therefore tested whether the yeast component of the diet could alter the feeding response to nutrient dilution, by comparing the effects on feeding frequency of DR using SYBrewer's yeast diet with that of a diet used in another published study, CSYExtract [15]. Similar to the data reported by Carvalho *et al.* (2005), we saw feeding frequency decrease as the concentration of CSYExtract in the medium was increased. In contrast, but consistent with previous reports [18], [19], flies feeding on the SYBrewer's diet under DR and full fed conditions did not change their feeding frequency. These data demonstrate that different DR recipes can elicit different behavioural responses. This is interesting because it may also mean that different diets affect lifespan-extension in different ways. The flies on SYBrewer's diet fed at the same frequency as flies subjected to DR conditions using CSYExtract, which suggests that flies on the full fed CSYExtract diet decrease their feeding to avoid higher concentrations of food. This is consistent with yeast extract having a toxic effect on flies and shortening lifespan [7].

An important element of studies into ageing is the longitudinal effects of lifespan-altering interventions. Previously, we have reported that flies subjected to DR do not alter their feeding frequency on day 7 of adult life [18]. It is still possible, however, that they do so later in life (day 40 onwards). We therefore conducted a longitudinal study of feeding frequency under DR. Very early in adult life (day 3) DR flies exhibited a higher feeding frequency than those under full fed conditions, but this did not occur over the majority of life and there were even individual instances of higher feeding frequency in full fed flies (later in life) than those subject to DR. This agrees with our previous longitudinal data on feeding frequency under DR [19]. This demonstrates that reduced nutrient intake does indeed correlate with extended lifespan for flies. Our data also show that the level of food consumption in older flies is remarkably lower in comparison to feeding levels in early-life (up to day 14), and more experiments will be required to understand how this lowered nutritional intake may contribute to declining mortality rates observed in late-life [38].

In recent years, various methods have been proposed to measure food intake in *Drosophila* [8], [12], [15]. However, none of these methods allow *Drosophila* to be measured during conditions that reflect either the experimental set-up they are normally housed in or their feeding frequency in undisturbed conditions. We established that the proboscis-extension method fulfils these criteria.

## Methods

### Fly stocks and dietary conditions

Wild type Dahomey flies were housed and maintained as described in Bass *et al.* (2007) [7]. The *chico^1^* allele is maintained as a balanced stock that has been backcrossed to the Dahomey outbred laboratory population as described in Clancy *et al.* (2001) [21]. *sn^w^*, *ry^506^*, *to^1^* (takeout) flies were a gift from Brigitte Dauwalder. All flies were maintained at 25°C, 65% humidity, on a 12h∶ 12h light∶ dark cycle. Unless stated otherwise, all assays used mated females at day 7 after eclosion. Day 7 was chosen because the flies are still young, but several early adult developmental processes have been completed [39]. All flies were reared for assays at a standard density, as for lifespan studies [40], and allowed to mate for 48 h post emergence before being sorted by sex, under light CO_2_ anaesthesia, into 30 mL glass vials containing 7 mL food.

The DR food medium contained 100 g autolysed Brewer's yeast powder (MP Biomedicals, Ohio, USA), 50 g sugar, 15 g agar, 30 ml nipagin (100 g/L), and 3 mL propionic acid made up to 1 litre of distilled water. The full fed food contained 200 g autolysed yeast powder, 50 g sugar, 15 g agar, 30 ml nipagin (100 g/L), and 3 ml propionic acid made up to 1 litre of distilled water [7]. In the diet comparison experiment, this medium is labelled SYBrewer's. CSYExtract was made according to [15]. This was made by co-diluting sugar and yeast extract (Bacto Yeast extract, B.D. Diagnostics, Sparks, MD) in a binder of cornmeal (80 g/L), bacto-agar (0.5%) and propionic acid (10 g/L). The 1× concentration contained 10 g/L sucrose and 10 g/L yeast extract.

For DR lifespan experiments, flies were maintained 5 per vial at 25°C, 65% humidity, on a 12h∶ 12h light∶ dark cycle. Proboscis-extension assays were performed for 60 minutes at 5-minute intervals, 4 hours after lights-on at 21 separate days across the lifespan experiment.

### Proboscis-extension assay during undisturbed conditions

For undisturbed observations of feeding, 7-day-old mated flies of the same sex, were transferred to new food at a density of 5 per vial on the evening before the assay. Flies were maintained in a pooled population, 100 flies per bottle, and a subset was collected and returned before and after the assay. Different measurements on different days were therefore considered to be independent of each other. Vials were coded and placed in a randomised order in rows on viewing racks at 25°C overnight. The assay occurred with minimal noise and physical disturbance to the flies. To avoid recording disturbed fly feeding behaviour, 30 minutes was allowed between the arrival of the observer and commencement of the assay. Observations were performed “blind” the next day for 90 minutes, commencing one hour after lights-on. In turn, each vial was observed for approximately 3 seconds during which the number of flies feeding was noted. A feeding event was scored when a fly had its proboscis extended and touching the food surface while performing a bobbing motion. Once all vials in the experiment had been scored in this way, successive rounds of observations were carried out in the same way for the whole 90 minutes of the assay, which, depending on the size of the experiment meant that each vial was observed once every 2 to 5 minutes. At the end of the assay, the vial labels were decoded and the feeding data expressed as a proportion by experimental group (sum of scored feeding events divided by total number of feeding opportunities, where total number of feeding opportunities = number of flies in vial×number of vials in the group×number of observations). For statistical analyses, comparisons between experimental groups were made on the totals of feeding events by all flies within a vial, to avoid pseudoreplication.

### Combined proboscis-extension and blue dye assay

Groups of five 7-day-old mated flies were transferred onto fresh food medium as indicated containing 2.5% (w/v) blue food dye (F D & C Blue Dye no. 1). Vials were scored approximately every 2 minutes for proboscis-extension and after a total of 30 minutes were transferred to eppendorf tubes and snap frozen in liquid nitrogen.

### Colour spectrophotometry

Flies were homogenised in 200 µL of distilled water. A further 800 µL of distilled water was added and the suspension passed through a 0.22 µm Millex filter (Millipore Corporation, Bedford) to remove debris and lipids. The absorbance of the liquid sample was then measured at 629 nm [Hitachi U-2001 Spectrophotometer (Lambda Advanced Technology Ltd., UK)]. Age-matched flies exposed to non-dyed food were used as the baseline during spectrophotometry. The amount of labelled food in the fly was calculated from a standard curve made by serial dilution in water of a sample of blue food.

### Statistics

Statistical analyses were performed using R, v2.2.1 [41]. To assess the relationship between proboscis-extensions and accumulation of blue dye, a linear mixed effects model was used. This modelled blue dye accumulation as a function of proportion of time observed feeding. Genotype, age and food concentration were specified as fixed effects and trial date as a random effect. To test for non-linearity, a quadratic term of observed feeding events was added to some models. The model fit for the data was reasonably acceptable, judging from residual plots and qq-plots (per trial date). For thoroughness, we re-analysed all models on log-transformed data. Although this further improved the normality of the residuals, the conclusions of the models were qualitatively unaffected.

To compare the effect of time of day, group size and dietary composition on feeding frequency, we used generalised linear models (with binomial error structure and logit link function, the deviances were scaled to correct for over-dispersion, and using *F*-tests for analysing significance). The generalised linear models incorporate information on the sample sizes and use weighted regression analyses. Significance among factor levels (e.g. among the 4 different group sizes) was determined by model simplification, where we evaluated whether combining >1 factor level into a single level led to a significant increase in deviance of the model, using *F*-tests [42]. The same generalised linear models were also used to compare the proportions of time spent feeding in the combined assays.

## References

[1] Gao X, Pan D (2001). TSC1 and TSC2 tumor suppressors antagonize insulin signaling in cell growth.. Genes Dev.

[2] Tapon N, Ito N, Dickson BJ, Treisman JE, Hariharan IK (2001). The *Drosophila* tuberous sclerosis complex gene homologs restrict cell growth and cell proliferation.. Cell.

[3] Ueno K, Ohta M, Morita H, Mikuni Y, Nakajima S (2001). Trehalose sensitivity in *Drosophila* correlates with mutations in and expression of the gustatory receptor gene Gr5a.. Curr Biol.

[4] Tompkins L, Cardosa MJ, White FV, Sanders TG (1979). Isolation and analysis of chemosensory behavior mutants in *Drosophila melanogaster*.. Proc Nat Acad Sci (USA).

[5] Ishimoto H, Matsumoto A, Tanimura T (2000). Molecular identification of a taste receptor gene for trehalose in *Drosophila*.. Science.

[6] Giannakou ME, Partridge L (2007). Role of insulin-like signalling in *Drosophila* lifespan.. Trends Biochem Sci.

[7] Bass TM, Grandison RC, Wong R, Martinez P, Partridge L (2007). Optimization of dietary restriction protocols in *Drosophila*.. J Gerontol A Biol Sci Med Sci.

[8] Ja WW, Carvalho GB, Mak EM, de la Rosa NN, Fang AY (2007). Prandiology of *Drosophila* and the CAFE assay.. Proc Nat Acad Sci (USA).

[9] Lee KP, Simpson SJ, Clissold FJ, Brooks R, Ballard JW (2008). Lifespan and reproduction in *Drosophila*: new insights from nutritional geometry.. Proc Nat Acad Sci (USA).

[10] Kimura M, Toda M, Beppu K, Watabe H (1977). Breeding sites of Drosophilid flies in and near Sapporo, northern Japan, with supplementary notes on adult feeding habits.. Jpn J Entomol.

[11] Carson HL, Lyon HL The ecology of *Drosophila* breeding sites..

[12] Edgecomb RS, Harth CE, Schneiderman AM (1994). Regulation of feeding behavior in adult *Drosophila melanogaster* varies with feeding regime and nutritional state.. J Exp Biol.

[13] Bross TG, Rogina B, Helfand SL (2005). Behavioral, physical, and demographic changes in *Drosophila* populations through dietary restriction.. Aging Cell.

[14] Min KJ, Tatar M (2005). *Drosophila* diet restriction in practice: do flies consume fewer nutrients?. Mech Ageing Dev.

[15] Carvalho GB, Kapahi P, Benzer S (2005). Compensatory ingestion upon dietary restriction in *Drosophila melanogaster*.. Nat Methods.

[16] Brummel T, Ching A, Seroude L, Simon AF, Benzer S (2004). *Drosophila* lifespan enhancement by exogenous bacteria.. Proc Nat Acad Sci (USA).

[17] King RC, Wilson LP (1955). Studies with Radiophosphorus in *Drosophila*.. J Exp Zool.

[18] Wong R, Piper MD, Blanc E, Partridge L (2008). Pitfalls of measuring feeding rate in the fruit fly *Drosophila melanogaster*.. Nat Methods.

[19] Mair W, Piper MD, Partridge L (2005). Calories do not explain extension of life span by dietary restriction in *Drosophila*.. PLoS Biol.

[20] Weindruch R, Walford R (1988). The retardation of aging and disease by dietary restriction.

[21] Clancy DJ, Gems D, Harshman LG, Oldham S, Stocker H (2001). Extension of life-span by loss of CHICO, a *Drosophila* insulin receptor substrate protein.. Science.

[22] Meunier N, Belgacem YH, Martin JR (2007). Regulation of feeding behaviour and locomotor activity by takeout in *Drosophila*.. J Exp Biol.

[23] Barnes AI, Wigby S, Boone JM, Partridge L, Chapman T (2008). Feeding, fecundity and lifespan in female *Drosophila melanogaster*.. Proc R Soc Lond B.

[24] Libert S, Zwiener J, Chu X, Vanvoorhies W, Roman G (2007). Regulation of *Drosophila* life span by olfaction and food-derived odors.. Science.

[25] Broughton SJ, Piper MD, Ikeya T, Bass TM, Jacobson J (2005). Longer lifespan, altered metabolism, and stress resistance in *Drosophila* from ablation of cells making insulin-like ligands.. Proc Nat Acad Sci (USA).

[26] Wu Q, Zhang Y, Xu J, Shen P (2005). Regulation of hunger-driven behaviors by neural ribosomal S6 kinase in *Drosophila*.. Proc Nat Acad Sci (USA).

[27] Oishi K, Shiota M, Sakamoto K, Kasamatsu M, Ishida N (2004). Feeding is not a more potent Zeitgeber than the light-dark cycle in *Drosophila*.. Neuroreport.

[28] Wertheim B, Allemand R, Vet LEM, Dicke M (2006). Effects of aggregation pheromone on individual behaviour and food web interactions: a field study on *Drosophila*.. Ecol Entomol.

[29] Chen S, Lee AY, Bowens NM, Huber R, Kravitz EA (2002). Fighting fruit flies: a model system for the study of aggression.. Proc Nat Acad Sci (USA).

[30] Mair W, Goymer P, Pletcher SD, Partridge L (2003). Demography of dietary restriction and death in *Drosophila*.. Science.

[31] Lints FA, Soliman MH (1988). *Drosophila* as a model organism for ageing studies.

[32] Oliver B, Perrimon N, Mahowald AP (1987). The ovo locus is required for sex-specific germ line maintenance in *Drosophila*.. Genes Dev.

[33] Partridge L, Gems D (2002). Mechanisms of ageing: public or private?. Nat Rev Genet.

[34] Kenyon C (2005). The plasticity of aging: insights from long-lived mutants.. Cell.

[35] Liang H, Masoro EJ, Nelson JF, Strong R, McMahan CA (2003). Genetic mouse models of extended lifespan.. Exp Gerontol.

[36] Partridge L, Gems D, Withers DJ (2005). Sex and death: what is the connection?. Cell.

[37] Clancy DJ, Gems D, Hafen E, Leevers SJ, Partridge L (2002). Dietary restriction in long-lived dwarf flies.. Science.

[38] Curtsinger JW, Fukui HH, Townsend DR, Vaupel JW (1992). Demography of genotypes: failure of the limited life-span paradigm in *Drosophila melanogaster*.. Science.

[39] Johnson MB, Butterworth FM (1985). Maturation and aging of adult fat body and oenocytes in *Drosophila* as revealed by light microscopic morphometry.. J Morphol.

[40] Kennington WJ, Gilchrist AS, Goldstein DB, Partridge L (2001). The genetic bases of divergence in desiccation and starvation resistance among tropical and temperate populations of *Drosophila melanogaster*.. Heredity.

[41] R: A language and environment for statistical computing (2005).

[42] Crawley MJ (2005). Statistics: An introduction using R.

